# Factors associated with lack of access to dental services among Brazilian adults

**DOI:** 10.11606/s1518-8787.2026060007431

**Published:** 2026-09-16

**Authors:** Márcio Vinicius de Gouveia Affonso, Aylene Bousquat, José Leopoldo Ferreira Antunes, Paulo Frazão

**Affiliations:** I Universidade de São Paulo Faculdade de Saúde Pública Departamento de Política, Gestão e Saúde São Paulo SP Brasil Universidade de São Paulo. Faculdade de Saúde Pública. Departamento de Política, Gestão e Saúde. São Paulo, SP, Brasil; II Universidade de São Paulo Faculdade de Saúde Pública Departamento de Epidemiologia São Paulo SP Brasil Universidade de São Paulo. Faculdade de Saúde Pública. Departamento de Epidemiologia. São Paulo, SP, Brasil

**Keywords:** Effective Access to Health Services, Equity in Access to Health Services, Oral Health Services, Oral Health Surveys, Socioeconomic Factors, Multilevel Analysis

## Abstract

**OBJECTIVE:**

To investigate the association between individual and contextual variables and lack of access to dental services among the Brazilian adult population.

**METHODS:**

This is a cross-sectional study based on a nationally representative sample of adults aged 35 to 44 years from SB Brasil 2023. Multiple analyses were performed using multilevel logistic regression. The outcome was lack of access to dental services. Individual exposures included demographic data, income, educational attainment, and type of service sought; contextual variables included population size, socioeconomic development, and availability of public dental services in Brazilian cities.

**RESULTS:**

It was found that 17.5% of Brazilian adults did not receive dental care when they sought it. Low educational attainment, low family income, and seeking public services increased the likelihood of lack of access. The contextual variables did not explain the outcome, in addition to the individual-level factors.

**CONCLUSION:**

Lack of access to dental services among adults in Brazil was associated with low socioeconomic status and with those who sought public services, considering contextual variables related to the municipalities included in the study. The absence of an association between the contextual variables analyzed and lack of access may reflect the widespread availability of dental services, an extremely positive aspect of the Brazilian health system. Nevertheless, despite advances in expanding coverage and the establishment of the Política Nacional de Saúde Bucal (National Oral Health Policy) as a state policy, socioeconomic inequalities persist that limit the principle of equitable access.

## INTRODUCTION

Access to health care is a complex and multidimensional concept that can be defined as the opportunity or ease with which users or communities are able to obtain appropriate care that meets their needs. User characteristics interact with factors related to institutions, organizations, and health care providers that refer to accessibility. Ensuring equitable access to health care services regardless of each user’s individual characteristics is a key priority for many health care systems and a topic on the global agenda^1^.

Access to and utilization of health services are interrelated, since utilization depends on access; however, they should not be understood as synonyms, as the actual utilization of a given service also depends on individual factors^2,4^. Although publications in the scientific literature may use measures of health service utilization as a proxy for access, it is important to consider that access precedes service use, and not the other way around^4,7,8^. Access to health services can be assessed through four dimensions: geographic accessibility, availability, financial accessibility, and acceptability^9^. These dimensions can be affected by individual and household characteristics, such as poverty and social vulnerability, which generally have a negative impact on access^9^.

In the Brazilian context specifically, progress must be acknowledged in ensuring universal access to health services following the implementation of the Sistema Único de Saúde (SUS – Unified Health System), even though limitations generally associated with socioeconomic factors remain^1^. The striking socioeconomic inequality that persists in the country exacerbates inequality in access to health care^10^.

A study conducted on a representative sample of the Brazilian population found that lack of access—defined as a request for dental care that was not met on the first attempt—ranged from 4.5% to 3.3% in 2003 and 2008. The disadvantage for the lowest *per capita* family income group was 11.0 and 7.7 times greater than that of the highest income group in the years mentioned. The rate of unmet dental care needs due to a lack of available appointments decreased by approximately 20.0% from 2003 to 2008^11^. These findings were similar to those reported in 1998^12^ and reinforce the importance of individuals’ socioeconomic status for access to dental services but provide no information on possible effects stemming from the type of service sought or contextual characteristics such as the availability of public dental services and the level of development of the user’s city of residence.

The Pesquisa Nacional de Saúde Bucal (National Oral Health Survey), conducted in 2023, included questions that allow for an analysis of access to dental services, rather than merely their utilization, as in previous editions. Respondents were asked whether, when seeking dental care, they actually received it. Approximately one-third of participants aged 35 to 44 reported not having sought dental services in the past year (36.0%), and those who sought care and were treated accounted for 42.5% of participants, with significant variations across Brazil’s geographic regions. Approximately 11.0% of Brazilian adults (aged 35 to 44) sought care but were not seen or scheduled for an appointment. However, no study has investigated the possible isolated or combined effects of individual and contextual characteristics on access to dental services.

Given the above, identifying potential factors related to access to oral health services in different Brazilian municipalities is important for broadening our understanding of this phenomenon. Thus, the objective was to investigate the association between individual and contextual variables and the lack of access to dental services among the Brazilian adult population.

## METHODS

### Study Design

This is a cross-sectional study that used data from the Pesquisa Nacional de Saúde Bucal—SB Brasil 2023. This survey assessed the oral health conditions of the Brazilian population in 2023 and is a household-based survey with a complex sample representative of Brazilians residing in permanent private households in urban areas throughout the country.

### Study Population

The SB Brasil 2023 sample was designed to be representative of the country as a whole, of each macroregion, and of each of the 53 domains corresponding to the 26 state capitals, the Federal District, and the 26 city clusters in the interior of each state. In each of these, census tracts and households were randomly selected. Further details on the sampling process have been described in other publications^13,14^. In the present study, the analysis was restricted to the adult age group, 35 to 44 years of age.

### Study Variables

The outcome was lack of access to dental care, defined by responses to the following question: “In the past year, did you seek any dental care?” The response categories were: 0) I did not seek care; 1) I sought care but was not seen; 2) I sought care and was scheduled for another day/location; 3) I sought care and was seen; 9) I don’t know/did not answer. Adults who sought care but were not seen (response 1) were compared to those who sought care and were either seen or scheduled for a later date (responses 2 and 3). The exclusion of individuals who did not seek dental care is justified because the absence of a request for care does not necessarily imply a barrier to access; it may stem from the user’s self-perception that they did not feel the need to consult a healthcare professional.

The demographic and socioeconomic characteristics included were: age (35 to 39 years and 40 to 44 years), sex (male and female), educational attainment (incomplete elementary school, complete elementary school and incomplete high school, complete high school, and incomplete or complete higher education), household income in terms of minimum wages (no information, up to one minimum wage, one to three minimum wages, and more than three minimum wages), participation in the government cash transfer program—the Programa Bolsa Família (PBF – Bolsa Família Program) (no, yes, and don’t know/did not answer), and type of service sought (public/private). Due to the low number of adults who self-identified as Asian (77) and Indigenous (26), both categories were grouped under “other.” Thus, the color/race variable was analyzed in four categories (white, mixed-race, Black, and other).

Taking into account the analytical model presented in the Figure, the municipal-level contextual variables included were: population size, obtained from the 2022 Demographic Census and divided into four categories (up to 20,000 inhabitants; > 20,000 to 50,000 inhabitants; > 50,000 to 100,000 inhabitants; and > 100,000 inhabitants); the Índice Firjan de Desenvolvimento Municipal (IFDM – FIRJAN Municipal Development Index) for the year 2023, a composite indicator that combines different aspects of economic dynamics, education, and health, with values grouped into four categories: up to 0.39 (critical level); 0.40 to 0.59 (low development); 0.60 to 0.79 (medium development) and 0.80 and above (high development)^15^; and the potential coverage of oral health teams in primary care, as reported by the Secretaria de Atenção Primária à Saúde (Secretariat of Primary Health Care) of the Ministry of Health for July 2023, which reflects the availability of dental services within the SUS primary health care network maintained by municipalities and was divided into three categories: up to 39.9%; 40.0% to 59.9%; and 60.0% and above.

**Figure:**
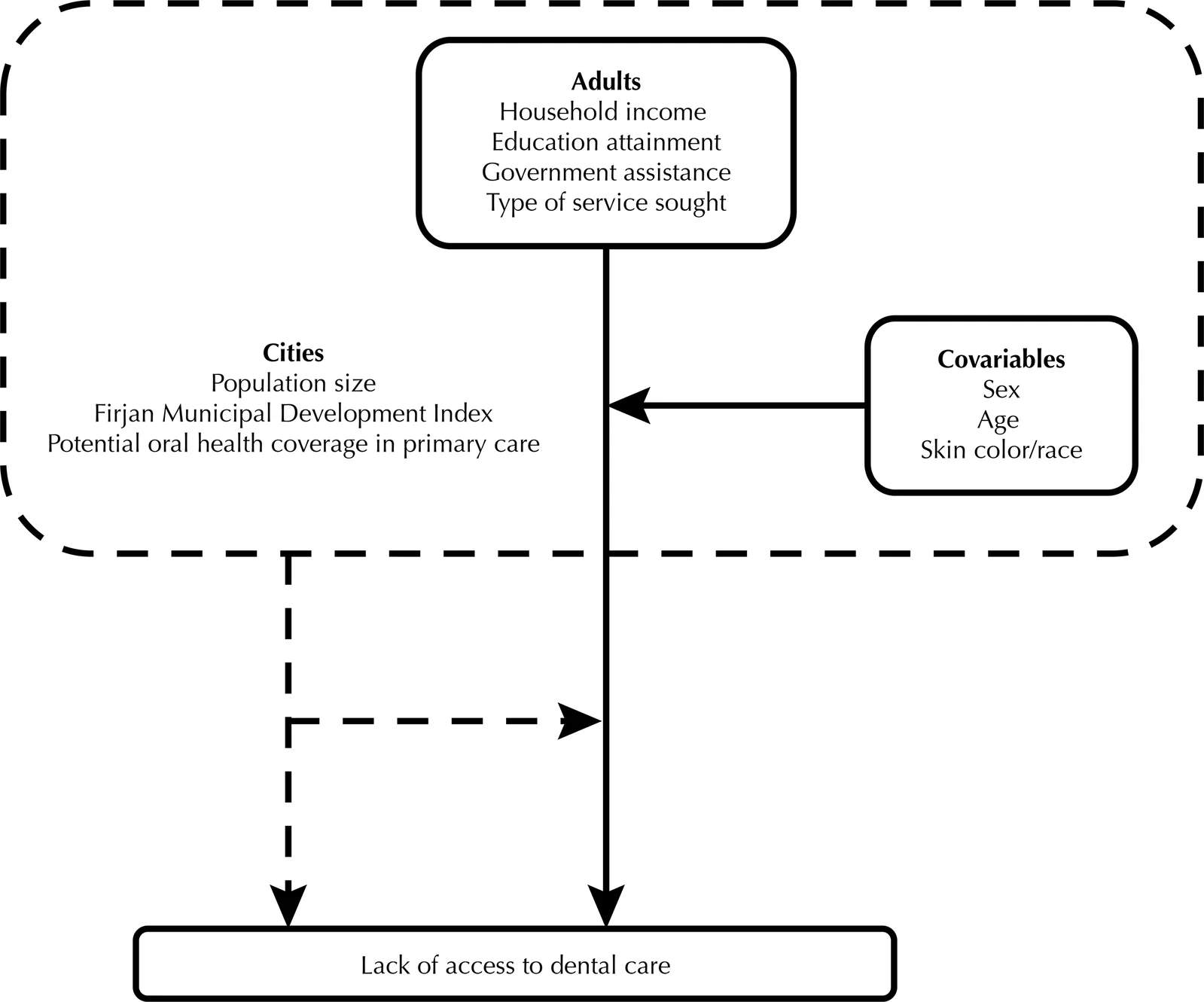
Model for analyzing the relationship between individual and contextual variables in the lack of access to dental services. Note: individual variables (solid line); contextual variables (dashed line).

The selection of these contextual variables is based on the understanding that the territory reflects socio-spatial relationships that influence access to health services, including dental services. According to Santos and Silveira^16^, territory is constituted by technical, scientific, and informational densities that determine its capacity to attract activities, resources, and people. Bousquat^17^highlights that the territorial division of labor and varying levels of urbanization shape the distribution of health technologies, which impacts access to health services.

In this sense, even though they are aggregated indicators at the municipal level, it is possible to analyze the distribution of dental services and the territorial characteristics associated with the availability of these services. While the demographic size reflects the location’s attractiveness accumulated over its history, likely as a result of long-standing public policies that favored the growth of various economic sectors, including dental services, and the IFDM synthesizes economic, educational, and health aspects of the municipality, the potential coverage of primary oral health care represents the availability of public dental services relative to the municipality’s population. Nationwide, more than 70.0% of municipalities have 60.0% or more potential coverage for oral health in primary care, with the majority distributed among municipalities with up to 100,000 inhabitants and those with medium, low, or critical levels of development.

### Data Analysis

The characteristics of the study population were described, considering the sample weight and the effect of the complex design on the sample stratification structure, using the instructions provided by the database curator and the survey package of the R software. The absolute and relative frequencies and their respective 95% intervals were described, including the population estimate.

Prior to the multilevel logistic regression analysis, multicollinearity among the individual variables was examined using the variance inflation factor (VIF) and tolerance (1/VIF) values. Next, associations were assessed between the outcome and each individual and contextual independent variable, in which adults (level 1) were grouped within cities (level 2). As a measure of association, the odds ratio (OR) and its unadjusted values were calculated. Those associations with a p-value < 0.20 were included in the multiple analysis.

An adjusted analysis was conducted to assess the contribution of each variable to the final model. Initially, a random intercept model without predictors (Model 0) was fitted to estimate the variance in the outcome across cities. Next, Model 1 included all variables with p < 0.20, excluding sex, age, and race/ethnicity, which were included as adjustment covariates. The aim was thus to operationalize the multilevel logistic regression by combining prior theoretical knowledge with the statistical potential of each variable. Models 2, 3, and 4 separately incorporated population size, socioeconomic development, and potential coverage of primary oral health care at the municipal level. For each model, the intraclass correlation coefficient was calculated to determine the magnitude of the variance among cities explained by the included variables. The likelihood ratio test with a 5% significance level was used to compare the models. Stata v.15.0 was used for statistical analysis.

## RESULTS

The sample analyzed in this study consisted of 5,338 Brazilian adults aged 35 to 44 years, representing an estimated population of 19,516,563 individuals in the country (Table 1). Regarding access to dental services, among adults who reported having sought care, 82.5% (95% CI 78.6–85.8) were seen or scheduled for an appointment, while 17.5% (95% CI 14.1–21.4) did not achieve this outcome, indicating a lack of access to dental services.

**Table 1:** Distribution of the Brazilian adult population aged 35 to 44 years according to study variables. Brazil, 2023.

Individual variables	(19,516,563) n^a^	(5,338) n^b^ (%)	Proportion (95% CI)
Outcome			
Sought care but was not seen	3,414,272	829 (16.0)	17.5 (14.2–21.4)
Sought care and received it	16,102,291	4,509 (84.0)	82.5 (78.6–85.8)
Gender			
Male	5,841,497	1,540 (29.0)	29.9 (26.6–33.5)
Female	13,675,066	3,798 (71.0)	70.1 (66.5–73.4)
Age			
35 to 39 years	10,138,274	2,657 (50.0)	51.9 (47.9–56.0)
40 to 44 years	9,378,289	2,681 (50.0)	48.1 (44.0–52.1)
Race/Ethnicity			
White	8,416,978	1,779 (33.0)	43.1 (39.0–47.3)
Brown	8,263,292	2,762 (52.0)	42.3 (38.2–46.6)
Black	2,521,005	694 (13.0)	12.2 (10.7–15.6)
Other	315,288	103 (2.0)	1.6 (1.0–2.7)
Programa Bolsa Família^c^			
No	14,560,610	3,627 (68.0)	74.6 (69.6–79.1)
Yes	3,920,342	1,428 (27.0)	20.1 (16.6–24.1)
Don’t know/did not answer	1,035,610	283 (5.0)	5.3 (2.8–9.8)
Type of service sought			
Public service	9,244,010	2,687 (50.0)	47.4 (41.9–52.9)
Private services	10,272,553	2,651 (40.0)	52.6 (47.1–58.1)
Education			
Incomplete elementary school	2,644,193	734 (14.0)	13.5 (11.2–16.2)
Complete elementary school and incomplete high school	3,841,540	891 (17.0)	19.7 (15.7–24.3)
Complete high school and incomplete higher education	9,069,458	2,450 (46.0)	46.5 (40.7–52.4)
Complete higher education	3,961,372	1,263 (24.0)	20.3 (16.8–24.3)
Household income in minimum wages (MW)			
No information	4,554,184	1,348 (25.0)	23.3 (18.4–29.1)
Up to 1 MW	3,691,039	981 (18.0)	18.9 (14.8–23.9)
> 1 to 3 MW	6,827,223	1,829 (34.0)	35.0 (30.0–40.3)
> 3 MW	4,444,117	1,180 (22.0)	22.8 (19.0–27.0)
**Municipal variables**			
Population size			
Up to 20,000		69 (21.5)	12.8 (8.4–19.1)
> 20,000 to 50,000		69 (21.5)	12.5 (8.5–18)
> 50,000 to 100,000		56 (17.5)	10.6 (6.8–16.1)
> 100,000		126 (39.5)	64.1 (56.0–71.4)
Socioeconomic development level			
Critical (< 0.4)		13 (4.0)	1.2 (0.5–2.8)
Low (0.4 to < 0.6)		128 (40.0)	23.4 (18.2–29.6)
Medium (0.6 to < 0.8)		145 (45.4)	52.0 (42.6–61.2)
High (≥ 0.8)		34 (10.6)	23.4 (16.7–31.8)
Potential oral health coverage in PC			
< 40%		53 (16.6)	30.9 (21.9–41.8)
40% to < 60%		64 (20.0)	29.8 (22.9–37.9)
≥ 60%		203 (63.4)	39.2 (31.6–47.4)

95% CI: 95% confidence interval; PC: primary care.

Note: The “Programa Bolsa Família” variable refers to participation in the government cash transfer program.

^a^ Equivalent to the population estimate.

^b^ Sample from the SB Brasil 2023 study (%).

^c^ Government conditional cash transfer program.

In the study population, there was a balance between the 35–39 (51.9%; 95% CI 47.8–56.0) and 40–44 (48.1%; 95% CI 44.0–52.1) age groups. Women predominated (70.1%; 95% CI 66.5–73.4), as did adults who self-identified as white (43.1%; 95% CI 39.0–47.3) and mixed-race (42.3%; 95% CI 38.2–46.6), as well as those who did not receive government assistance (74.6%; 95% CI 69.6–79.1). Nearly half of the sample (46.5%; 95% CI 40.7–52.4) had complete high school or had not completed higher education, and regarding household income, 35.0% (95% CI 30.0–40.3) earned between one and three minimum wages. Regarding the type of service sought, 47.4% (95% CI 41.9–52.9) sought care at a public facility and 52.6% (95% CI 47.1–58.1) at a private facility.

Table 2 shows the unadjusted analysis. Lower levels of education and income, participation in the PBF, and seeking public dental care were significantly associated with a higher likelihood of lack of access to dental services. Adults who had completed elementary school or had not completed high school were nearly three times more likely to lack access (OR = 2.87; 95% CI 2.21–3.72; p < 0.000) compared to those who had completed higher education. A similar finding was observed when comparing adults with a household income of up to one minimum wage to those with an income exceeding three minimum wages (OR = 2.36; 95% CI 1.80–3.08; p = 0.000). Individuals enrolled in the PBF were 26% more likely to lack access (OR = 1.26; 95% CI 1.05–1.50; p = 0.009) compared to non-beneficiaries. Those who sought public dental care were more than twice as likely to lack access (OR = 2.25; 95% CI 1.91–2.66; p < 0.000) compared to those who sought private care. There was no significant association between the outcome and the variables age (p = 0.474), sex (p = 0.816), and categories of skin color/race.

**Table 2:** Unadjusted multilevel logistic regression for lack of access to dental services according to individual and contextual variables in the Brazilian adult population aged 35 to 44 years. Brazil, 2023 (5,338 adults in 320 cities).

Individual variables	Sought care but was not seen or scheduled				
	n	%	OR	95% CI	p
Gender					
Male	237	16.8	Ref.		
Female	592	17.8	1.02	0.86–1.20	0.816
Age					
35 to 39 years	404	19.2	Ref.		
40 to 44 years	425	15.7	1.05	0.90–1.23	0.474
Race/Ethnicity					
White	251	18.3	Ref.		
Brown	446	17.4	1.07	0.89–1.29	0.495
Black	112	14.7	1.16	0.90–1.50	0.261
Other	20	20.6	1.41	0.83–2.39	0.204
Programa Bolsa Família					
No	514	16.2	Ref.		
Yes	246	20.1	**1.26**	**1.05–1.50**	**0.009**
Don’t know/did not answer	69	25.3	**1.70**	**1.23–2.34**	**0.001**
Type of service Sought					
Private	274	16.2	Ref.		
Public	555	18.9	**2.25**	**1.91–2.66**	**< 0.000**
Education					
Incomplete elementary school	115	8.8	Ref.		
Complete elementary school and incomplete high school	380	18.6	**1.85**	**1.47–2.32**	**< 0.000**
Complete high school and incomplete higher education	197	23.5	**2.87**	**2.21–3.72**	**< 0.000**
Complete higher education	137	18	**2.55**	**1.92–3.37**	**< 0.000**
Household income in minimum wages (MW)					
> 3 MW	117	10.4	Ref.		
> 1 to 3 MW	285	13.5	**1.65**	**1.30–2.10**	**< 0.000**
Up to 1 MW	188	26.7	**2.36**	**1.80–3.08**	**< 0.000**
No information	239	22.9	**1.88**	**1.46–2.42**	**< 0.000**
**Municipal variables**					
Population size					
> 100,000	126	16.1	Ref.		
> 50,000 to 100,000	56	17.6	1.13	0.70–1.82	0.604
> 20,000 to 50,000	69	17.5	1.24	0.82–1.88	0.290
Up to 20,000	69	24.2	1.34	0.87–2.06	0.175
Socioeconomic development level					
High (≥ 0.8)	34	12.2	Ref.		
Medium (0.6 to < 0.8)	145	19.5	1.47	0.89–2.43	0.126
Low (0.4 to < 0.6)	128	17.4	1.49	0.88–2.54	0.135
Critical (< 0.4)	13	33.6	1.91	0.77–4.75	0.158
Potential oral health coverage in PC					
≥ 60%	203	19.5	Ref.		
40% to < 60%	64	9.7	**0.68**	**0.47–0.98**	**0.040**
< 40%	53	22.4	1.05	0.70–1.58	0.779

OR: odds ratio; 95% CI: 95% confidence interval; PC: primary care.

Note: The “Programa Bolsa Família” variable refers to participation in the government cash transfer program. Bold values represent significant associations.

Regarding contextual variables, only oral health coverage in primary care between 40.0% and 60.0% was associated with a lower likelihood of lack of access (OR = 0.68; 95% CI 0.47–0.98; p = 0.040) compared to coverage of 60.0% or higher. The other contextual variables did not show statistically significant associations (Table 2). When examining the characteristics of the municipalities where the study population resided, a significant association was observed between municipalities with more than 100,000 inhabitants and primary care oral health coverage rates below 60.0% (p < 0.000), and between municipalities with low and critical levels of development and primary care oral health coverage rates of 60.0% or higher (p < 0.000) (data not shown in Table 2).

In the adjusted multilevel model (Table 3), the associations between lack of access, educational attainment, household income, and type of service sought remained significant, even after adjusting for sex and age (Model 1). Adults who had not completed elementary school were 1.75 (95% CI 1.28–2.38) times more likely to lack access to dental services compared to individuals who had completed higher education. This likelihood was 2.10 (95% CI 1.59–2.79) times higher for adults who had completed elementary school but not high school. Regarding income, individuals with a household income below the minimum wage were 1.51 (95% CI 1.12–2.04) times more likely to experience a lack of access. Adults who sought public dental services were nearly twice as likely to be unable to access care (OR = 1.90; 95% CI 1.59–2.26).

**Table 3:** Multilevel logistic regression adjusted for lack of access to dental services according to individual and contextual variables in the Brazilian adult population aged 35 to 44 years. Brazil, 2023 (5,338 adults in 320 cities).

Fixed effects	Model 1^a^	Model 2^a^	Model 3^a^	Model 4^a^
Individual variables	OR (95% CI)	OR (95% CI)	OR (95% CI)	OR (95% CI)
Bolsa Família Program				
No	Ref.	Ref.	Ref.	Ref.
Yes	0.88 (0.73–1.07)	0.88 (0.73–1.07)	0.90 (0.73–1.08)	0.89 (0.73–1.08)
Don’t know/did not answer	1.26 (0.90–1.77)	1.26 (0.90–1.78)	1.26 (0.89–1.77)	1.26 (0.89–1.77)
Type of service sought				
Private	Ref.	Ref.	Ref.	Ref.
Public	**1.90 (1.60–2.26)**	**1.90 (1.59–2.26)**	**1.90 (1.58–2.27)**	**1.92 (1.61–2.28)**
Education				
Complete higher education	Ref.	Ref.	Ref.	Ref.
Complete high school and incomplete higher education	**1.46 (1.15–1.87)**	**1.47 (1.16–1.88)**	**1.47 (1.15–1.87)**	**1.46 (1.15–1.87)**
Complete elementary school and incomplete high school	**2.12 (1.60–2.82)**	**2.14 (1.61–2.83)**	**2.13 (1.61–2.83)**	**2.13 (1.61–2.82)**
Incomplete elementary school	**1.75 (1.28–2.39)**	**1.75 (1.28–2.38)**	**1.76 (1.29–2.40)**	**1.76 (1.29–2.40)**
Household income in minimum wages				
> 3 MW	Ref.	Ref.	Ref.	Ref.
> 1 to 3 MW	1.22 (0.94–1.58)	1.22 (0.94–1.57)	1.22 (0.94–1.57)	1.23 (0.95–1.58)
Up to 1 MW	**1.52 (1.13–2.05)**	**1.51 (1.12–2.04)**	**1.51 (1.12–2.03)**	**1.52 (1.13–2.05)**
No information	**1.35 (1.02–1.77)**	**1.35 (1.03–1.77)**	**1.35 (1.02–1.77)**	**1.33 (1.01–1.75)**
**Municipal variables**				
Population size				
> 100,000		Ref.		
> 50,000 to 100,000		1.11 (0.69–1.78)		
> 20,000 to 50,000		1.21 (0.80–1.83)		
Up to 20,000		1.14 (0.74–1.74)		
Socioeconomic development level				
High (≥ 0.8)			Ref.	
Medium (0.6 to < 0.8)			1.34 (0.82–2.18)	
Low (0.4 to < 0.6)			1.21 (0.71–2.05)	
Critical (< 0.4)			1.52 (0.61–3.75)	
Potential oral health coverage in PC				
≥ 60%				Ref.
40% to < 60%				0.73 (0.51–1.04)
< 40%				1.22 (0.81–1.83)
Random effects	Estimates			
Variance (standard error)	0.405 (0.150)	0.390 (0.146)	0.409 (0.150)	0.420 (0.148)
Empty model 0.483 (0.163)				
Percentage of variance explained	16.1%	3.2%	-0.8%	-3.1%
Likelihood ratio test	< 0.000	< 0.000	< 0.000	< 0.000
ICC	16.1	19.3	15.3	13.0

OR: odds ratio; 95% CI: 95% confidence interval; PC: primary care; MW: minimum wage; ICC: intraclass correlation coefficient.

Note: ICC corresponds to the percentage of total variance at level 2 explained by the model; the “Programa Bolsa Família” variable refers to participation in the government cash transfer program. Bold values represent significant associations.

^a^ Adjusted for demographic variables (sex, age, and race/ethnicity).

Among the municipal variables, no significant associations were observed in the final model. The OR values for municipalities with intermediate oral health coverage in primary care (40.0% to 60.0%) lost statistical significance after adjustment for individual variables (OR = 0.73; 95% CI 0.51–1.04; p = 0.079) compared to municipalities with higher coverage (greater than or equal to 60.0%). The variance among the studied municipalities decreased from 0.48 in the null model to 0.41 in Model 1 and 0.39 in Model 2. The inclusion of population size explained 19.3% of the total variance.

## DISCUSSION

The results showed that socioeconomic status, as reflected by low levels of education and family income, and the use of public services were significantly associated with a lack of access to dental services among Brazilian adults, considering their distribution across cities, after adjusting for sociodemographic factors and the characteristics of the municipalities of residence related to population size, socioeconomic development, and potential coverage of dental services in the primary health care system. Contextual factors did not explain the outcome in addition to individual-level factors. This result is also relevant because it is consistent with the hypothesis of the widespread availability of dental services across the country’s different municipalities.

Unlike the literature on characteristics linked to the use of health services, there is a lack of studies investigating factors associated with access to these services^6^. The multiplicity of definitions and interpretations of the term and the frequent use of the concept of utilization as a proxy for access may be linked to this gap. With a greater focus on individual characteristics, Nunes et al.^18^ observed that lack of access was more frequent among individuals with lower income, but the same pattern was not observed when service utilization was assessed. This finding serves as a warning regarding the conceptual differences between the terms and reinforces the importance of studies that treat access as a distinct and multifactorial phenomenon, as proposed by this study.

The unfavorable results for adults with lower income and educational attainment were consistent with two Brazilian population-based studies^11,19^. The observed discrepancies relate to estimates of lack of access and can be attributed primarily to differences in the age of the population under study and the indicator used. While in the present study, the rate was 17.5% among adults aged 35 to 44 who sought care but were not seen or scheduled an appointment in the past 12 months, the study using data from a 2009 telephone survey reported a rate of 15.2% for the same indicator, but the population aged 18 and older residing in the 26 state capitals and the Federal District^19^, and in the study using data from the Pesquisa Nacional por Amostra de Domicílios (National Household Sample Survey) for the years 2003 and 2008, rates of 4.5% and 3.3%, respectively, were reported, considering only the last two weeks and all age groups^11^.

Chaves et al.^20^ and Carreiro et al.^21^ investigated the lack of access to dental services in municipalities in the states of Bahia and Minas Gerais, respectively. A lack of access was reported by 15.2% of individuals in the municipalities in Bahia and by 10.3% in the municipality in Minas Gerais^21^. While Chaves et al.^20^ defined access as synonymous with accessibility, that is, characteristics of the services that facilitate their use by patients, Carreiro et al.^21^ defined access as the receipt of care in response to an individual’s request for assistance.

According to Almeida et al.^6^, universal health care systems, such as the SUS, hold great potential for reducing inequities in access to health care services. However, in the present study, a higher likelihood of lack of access was observed among adults who sought care through the public health system. One might hypothesize that when adults seek dental care at public facilities, they are exposed to services with organizational characteristics that are incompatible with their needs (incompatible shifts and/or operating hours, long lines, low efficiency, and geographic locations that are difficult to access)^22^. In this regard, Chaves et al.^20^ recommended several strategies for scheduling appointments, which could help replace no-shows and reduce waiting lines for care. Furthermore, a study conducted in more than 18,000 public facilities in 2014 showed that only 14.8% of them had all the necessary supplies to perform primary care dental procedures^23^. For that same year, there was one dentist affiliated with the SUS for approximately 4,000 people, while in the private sector, the ratio was 1 to 649 inhabitants^24^.

In addition to these factors related to the public health system, the socioeconomic and structural conditions of the areas where such services are located could also be associated with access among the adult population^17,25^. Factors related to transportation and the low availability of dental services in certain localities could act as barriers limiting access to these services^26^. However, the observed results were consistent with the hypothesis of widespread availability of dental services across the country’s different municipalities; that is, the heterogeneity that characterizes municipalities in terms of population size, socioeconomic development, and the provision of public dental services at the primary care level was not significant enough to reflect relevant differences in access among Brazilian adults.

Among the study’s limitations, the inclusion of the PBF and household income variables with missing data in the analysis could have been raised as an issue. Since research on contextual factors related to access is scarce, and removing these observations could have reduced the number of cities included, the decision was made to treat these observations as a separate category to preserve the variety of level 2 observations and the interpretability of the findings. The type of access measure represented by self-report could be viewed as a limitation if the study’s focus were directed toward the characteristics of dental service provision. However, since the focus was on investigating access from the perspective of the urban adult population’s experience, the in-home interview can be considered the most appropriate data collection technique. Added to this are the methodological rigor and instrument validation that characterized the study^13^. The study design does not allow for interpreting the observed associations as cause-and-effect relationships or extrapolating the findings to the adult population residing in rural areas. Determining such relationships requires other types of studies and variables. For example, studies that include organizational variables linked to the characteristics of service provision may help elucidate the role these characteristics play in facilitating or hindering access among the adult population. Despite these limitations, it is worth highlighting the relevance of this study in analyzing the lack of access to dental services—defined as the failure to obtain care after identifying a need and seeking services—based on a complex population-based sample from the Pesquisa Nacional SB Brasil 2023 (2023 SB Brasil National Survey). Although the concept may take on different meanings in the scientific literature, a common aspect is that access, whether to a service, a service provider, or an institution, is defined in relation to the individual’s needs^1^. Another key point worth mentioning was the use of multilevel analysis, which allowed us to understand access to dental services based on the interaction between individual and contextual determinants, considering the socioeconomic and territorial characteristics of the municipalities—a perspective that has been little studied.

In conclusion, the lack of access to dental services among adults was associated with low socioeconomic status and with seeking public dental care, considering differences among municipalities related to population size, development, and the potential coverage of dental services within the primary health care network. The absence of an association between the contextual variables analyzed and the lack of access may reflect the widespread availability of dental services, an extremely positive aspect of the Brazilian health system. Nevertheless, despite advances in expanding coverage and the establishment of the Política Nacional de Saúde Bucal (National Oral Health Policy) as a state policy, socioeconomic inequalities persist that limit the principle of equitable access.
