# Supplementary material for: Factors associated with lack of access to dental services among Brazilian adults
Source: Rev Saude Publica. 2026 Sep 16;60:e45. doi: 10.11606/s1518-8787.2026060007431 (PMC13618993; doi:10.11606/s1518-8787.2026060007431)
Supplement: Translation

# Translation: Fatores associados à falta de acesso aos serviços odontológicos por adultos brasileiros

**Authors:** Márcio Vinicius de Gouveia Affonso, Aylene Bousquat, José Leopoldo Ferreira Antunes, Paulo Frazão

**Affiliations:** I Brasil Universidade de São Paulo. Faculdade de Saúde Pública. Departamento de Política, Gestão e Saúde. São Paulo, SP, Brasil; II Brasil Universidade de São Paulo. Faculdade de Saúde Pública. Departamento de Epidemiologia. São Paulo, SP, Brasil

**Keywords:** Acesso Efetivo aos Serviços de Saúde, Equidade no Acesso aos Serviços de Saúde, Serviços de Saúde Bucal, Inquéritos de Saúde Bucal, Fatores Socioeconômicos, Análise Multinível

## Abstract

**OBJETIVO:**

Investigar a associação de variáveis individuais e contextuais com a falta de acesso aos serviços odontológicos pela população adulta brasileira.

**MÉTODOS:**

Trata-se de um estudo transversal, com base em amostra representativa nacional de indivíduos adultos de 35 a 44 anos, proveniente do SB Brasil 2023. Foram feitas análises múltiplas por meio de regressão logística multinível. O desfecho foi a falta de acesso aos serviços odontológicos. As exposições individuais foram dados demográficos, renda, escolaridade, tipo de serviço procurado, e as variáveis contextuais foram porte populacional, desenvolvimento socioeconômico e disponibilidade de serviço público odontológico em cidades brasileiras.

**RESULTADOS:**

Identificou-se que 17,5% dos adultos brasileiros não obtiveram atendimento odontológico quando procuraram. Baixo nível de escolaridade, de renda familiar e procura por serviço público aumentaram a chance de falta de acesso. As variáveis contextuais não explicaram o desfecho, adicionalmente aos fatores de ordem individual.

**CONCLUSÃO:**

A falta de acesso aos serviços odontológicos no Brasil em adultos mostrou associação com a baixa condição socioeconômica e com aqueles que procuraram serviço público, considerando variáveis contextuais relacionadas aos municípios do estudo. A ausência de associação entre as variáveis contextuais analisadas e a falta de acesso pode refletir a capilaridade dos serviços odontológicos, um aspecto extremamente positivo do sistema de saúde brasileiro. Ainda assim, a despeito dos avanços na ampliação da cobertura e da instituição da Política Nacional de Saúde Bucal como política de Estado, persistem desigualdades socioeconômicas que limitam o princípio da equidade de acesso.

## INTRODUÇÃO

O acesso aos serviços de saúde é um conceito complexo e multidimensional que pode ser definido como a oportunidade ou facilidade com que usuários ou comunidades conseguem cuidados apropriados compatíveis com suas necessidades. As características dos usuários interagem com fatores relacionados a instituições, organizações e provedores de saúde que dizem respeito à acessibilidade. Assegurar equidade de acesso aos serviços de saúde independentemente das características individuais de cada usuário é uma importante prioridade de muitos sistemas de saúde e um tema da agenda global^1^.

Acesso e utilização de serviços de saúde estão inter-relacionados, uma vez que a utilização é dependente do acesso, entretanto, não devem ser entendidos como sinônimos, pois a utilização efetiva de um dado serviço depende, também, de aspectos individuais^2,4^. Ainda que possam ser encontradas publicações na literatura científica com medidas de utilização dos serviços de saúde como *proxy* para o acesso, é importante considerar que o acesso antecede o uso do serviço, e não o contrário^4,7,8^. O acesso aos serviços de saúde pode ser avaliado por meio de quatro dimensões: acessibilidade geográfica, disponibilidade, acessibilidade financeira e aceitabilidade^9^. Essas dimensões podem ser afetadas por características individuais e domiciliares, como a pobreza e a vulnerabilidade social, o que geralmente contribui negativamente ao acesso^9^.

No contexto brasileiro especificamente, há de se reconhecer avanços na garantia do acesso universal aos serviços de saúde a partir da implementação do Sistema Único de Saúde (SUS), ainda que limitações geralmente associadas a fatores socioeconômicos permaneçam^1^. A marcante desigualdade socioeconômica que persiste no país acentua a desigualdade no acesso à saúde^10^.

Estudo realizado em amostra representativa da população brasileira apontou que a falta de acesso – entendida como procura por serviço odontológico que não foi atendida na primeira tentativa – variou de 4,5% para 3,3% nos anos de 2003 e 2008. A desvantagem para o menor nível de renda familiar *per capita* foi 11,0 e 7,7 vezes maior em relação ao maior nível de renda nos anos mencionados. O não atendimento odontológico devido à falta de vagas diminuiu cerca de 20,0% de 2003 a 2008^11^. Estes achados foram similares aos encontrados em 1998^12^ e reforçam a importância da condição socioeconômica dos indivíduos para o acesso aos serviços odontológicos, mas nada informam sobre possível efeito decorrente do tipo de serviço procurado e de características contextuais como a disponibilidade de serviços públicos odontológicos e o grau de desenvolvimento da cidade de residência do usuário.

A Pesquisa Nacional de Saúde Bucal, realizada no ano de 2023, trouxe questões que permitem a análise do acesso aos serviços odontológicos, e não somente sua utilização como nas edições anteriores. Os entrevistados responderam se, ao procurarem atendimento odontológico, de fato o obtiveram. Aproximadamente um terço dos participantes de 35 a 44 anos relatou não ter procurado serviços odontológicos no último ano (36,0%), e os que procuraram e foram atendidos corresponderam a 42,5% dos participantes, com importantes variações entre as regiões geográficas brasileiras. Aproximadamente 11,0% dos adultos brasileiros (com idades entre 35 e 44 anos) procuraram atendimento e não foram atendidos ou agendados. Entretanto, nenhuma pesquisa investigou possíveis efeitos isolados ou combinados decorrentes das características individuais e contextuais sobre o acesso aos serviços odontológicos.

Diante do exposto, identificar possíveis fatores relacionados ao acesso aos serviços de saúde bucal em diferentes municípios brasileiros é relevante para ampliar a compreensão sobre o fenômeno. Assim, o objetivo foi investigar a associação de variáveis individuais e contextuais com a falta de acesso aos serviços odontológicos pela população adulta brasileira.

## MÉTODOS

### Desenho do Estudo

Trata-se de estudo transversal que utilizou dados da Pesquisa Nacional de Saúde Bucal – SB Brasil 2023. Esta pesquisa avaliou as condições de saúde bucal da população brasileira no ano de 2023 e se configura como um inquérito de base domiciliar, com amostragem complexa representativa de brasileiros residentes em domicílios particulares permanentes em área urbana de todo o território nacional.

### População do Estudo

A amostra do SB Brasil 2023 foi dimensionada para ser representativa do país como um todo, de cada macrorregião e de cada um dos 53 domínios correspondentes às 26 capitais das Unidades Federativas, ao Distrito Federal, e aos 26 agrupamentos de cidades do interior de cada estado. Em cada, foram sorteados setores censitários e domicílios. Mais detalhes sobre o processo de amostragem foram descritos em outras publicações^13,14^. No presente estudo, a análise foi restrita ao grupo etário adulto, de 35 a 44 anos de idade.

### Variáveis do Estudo

O desfecho foi a falta de acesso ao serviço odontológico, definida pelas respostas à seguinte pergunta: ”No último ano, procurou algum serviço odontológico?”. As categorias de resposta foram: 0) Não procurei; 1) Procurei e não fui atendido; 2) Procurei e fui agendado para outro dia/outro local; 3) Procurei e fui atendido; 9) Não sei/não respondeu. Os adultos que procuraram e não foram atendidos (resposta 1) foram comparados àqueles que procuraram e conseguiram atendimento ou agendamento (respostas 2 e 3). A exclusão dos indivíduos que não buscaram atendimento odontológico justifica-se porque a ausência de procura não implica, obrigatoriamente, barreira de acesso, podendo decorrer da autopercepção do usuário que não sentiu necessidade de consultar um profissional de saúde.

As características demográficas e socioeconômicas incluídas foram: idade (35 a 39 anos e 40 a 44 anos), sexo (masculino e feminino), escolaridade (ensino fundamental incompleto, ensino fundamental completo e médio incompleto, ensino médio completo e ensino superior incompleto e ensino superior completo), renda familiar em salários mínimos (sem informação, até um salário mínimo, de um a três salários mínimos e mais que três salários mínimos), participação em programa governamental de transferência de renda – Programa Bolsa Família (PBF) (não, sim e não sabe/não respondeu) e tipo de serviço procurado (público/privado). Devido à baixa frequência de adultos autodeclarados amarelos (77) e indígenas (26), ambas as categorias foram agrupadas na categoria ”outras”. Assim, a variável cor/raça foi analisada em quatro categorias (branca, parda, preta e outras).

Levando em consideração o modelo de análise apresentado na Figura, as variáveis contextuais de nível municipal incluídas foram: porte demográfico, obtido com base no Censo Demográfico de 2022, e separado em quatro categorias (até 20 mil habitantes; > 20 a 50 mil habitantes; > 50 a 100 mil habitantes e > 100 mil habitantes); índice Firjan de Desenvolvimento Municipal (IFDM) para o ano de 2023, um indicador composto que combina diferentes aspectos da dinâmica econômica, da educação e da saúde, cujos valores estão agrupados em quatro categorias: até 0,39 (nível crítico); 0,40 a 0,59 (baixo desenvolvimento); 0,60 a 0,79 (médio desenvolvimento) e 0,80 e mais (alto desenvolvimento)^15^; e a cobertura potencial de equipes de saúde bucal na atenção básica, disponibilizada pela Secretaria de Atenção Primária à Saúde do Ministério da Saúde para julho de 2023, que reflete a disponibilidade de serviços odontológicos da rede de atenção básica à saúde do SUS mantida pelos municípios e foi dividida em três categorias: até 39,9%; de 40,0% a 59,9% e de 60,0% e mais.

**Figura:** Modelo de análise da relação entre variáveis individuais e contextuais na falta de acesso aos serviços odontológicos. Nota: variáveis individuais (linha cheia); variáveis contextuais (linha tracejada).

A seleção destas variáveis contextuais se fundamenta na compreensão de que o território expressa relações socioespaciais que influenciam o acesso aos serviços de saúde, inclusive os odontológicos. Para Santos e Silveira^16^, o território é constituído por densidades técnicas, científicas e informacionais que determinam sua capacidade de atrair atividades, recursos e também pessoas. Bousquat^17^ destaca que a divisão territorial do trabalho e os distintos níveis de urbanização moldam a distribuição das tecnologias em saúde, o que impacta o acesso aos serviços de saúde.

Nesse sentido, ainda que se configurem como indicadores agregados ao nível municipal, é possível analisar a distribuição dos serviços odontológicos e as características territoriais associadas à disponibilidade desses serviços. Enquanto o porte demográfico reflete o poder de atração do local acumulado ao longo de sua história, provavelmente como efeito de políticas públicas de longa duração que favoreceram o crescimento de diversos setores econômicos, incluindo o de serviços odontológicos, e o IFDM sintetiza aspectos econômicos, educacionais e de saúde do município, a cobertura potencial da atenção básica em saúde bucal representa a disponibilidade de serviços públicos odontológicos em relação à população do município. No país, mais de 70,0% dos municípios têm 60,0% ou mais de cobertura potencial de saúde bucal na atenção básica, sendo que a maioria se distribui entre os municípios com até 100 mil habitantes e os municípios com desenvolvimento médio, baixo ou crítico.

### Análise dos Dados

Realizou-se a descrição das características da população do estudo considerando o peso amostral e o efeito do desenho complexo na estrutura de estratificação da amostra, com o auxílio das instruções fornecidas pelo curador do banco de dados e do pacote *survey* do *software* R. As frequências absoluta e relativa e respectivos intervalos de confiança de 95% (IC95%) foram descritos incluindo a estimativa populacional.

Previamente à análise de regressão logística multinível, a colinearidade entre as variáveis individuais foi examinada por meio dos valores do fator de inflação da variância (VIF) e de tolerância (1/VIF). Em seguida, foram avaliadas as associações entre o desfecho e cada variável independente individual e contextual, na qual os adultos (nível 1) foram agrupados dentro das cidades (nível 2). Como medida de associação, foram calculados os valores de *odds ratio* (OR) e respectivos IC95% não ajustados. Aquelas relações que apresentaram valor de p < 0,20 foram incluídas na análise múltipla.

Procedeu-se à análise ajustada para verificar a contribuição de cada variável no modelo final. Inicialmente, um modelo de intercepto aleatório sem preditores (Modelo 0) foi ajustado para estimar a variância do desfecho entre as cidades. Em seguida, o Modelo 1 incluiu todas as variáveis com p < 0,20, excetuando-se sexo, idade e cor/raça, incluídas como covariáveis de ajuste. Buscou-se, dessa forma, a operacionalização da regressão logística multinível a partir da combinação de conhecimento teórico prévio e do potencial estatístico de cada variável. Os Modelos 2, 3 e 4 incorporaram separadamente o porte populacional, o desenvolvimento socioeconômico e a cobertura potencial da atenção básica de saúde bucal em nível municipal. Para cada modelo, o coeficiente de correlação intraclasse foi calculado para determinar a magnitude da variância entre as cidades que foi explicada pelas variáveis incluídas. O teste da razão de verossimilhança com nível de significância de 5% foi adotado para comparar os modelos. O Stata v.15.0 foi usado na análise estatística.

## RESULTADOS

A amostra analisada neste estudo foi composta por 5.338 adultos brasileiros de 35 a 44 anos de idade, o que representa uma população estimada de 19.516.563 indivíduos no país (Tabela 1). Em relação ao acesso aos serviços odontológicos, dos adultos que relataram ter procurado um serviço, 82,5% (IC95% 78,6–85,8) foram atendidos ou agendados, enquanto 17,5% (IC95% 14,1–21,4) não obtiveram esse desfecho, configurando a falta de acesso aos serviços odontológicos.

**Tabela 1:** Distribuição da população de adultos brasileiros de 35 a 44 anos de idade segundo as variáveis do estudo. Brasil, 2023.

Variáveis individuais	(19.516.563) n^a^	(5.338) n^b^ (%)	Proporção (IC95%)
Desfecho			
Procurou e não foi atendido	3.414.272	829 (16,0)	17,5 (14,2–21,4)
Procurou e foi atendido	16.102.291	4.509 (84,0)	82,5 (78,6–85,8)
Sexo			
Masculino	5.841.497	1.540 (29,0)	29,9 (26,6–33,5)
Feminino	13.675.066	3.798 (71,0)	70,1 (66,5–73,4)
Idade			
35 a 39 anos	10.138.274	2.657 (50,0)	51,9 (47,9–56,0)
40 a 44 anos	9.378.289	2.681 (50,0)	48,1 (44,0–52,1)
Cor/raça			
Branca	8.416.978	1.779 (33,0)	43,1 (39,0–47,3)
Parda	8.263.292	2.762 (52,0)	42,3 (38,2–46,6)
Preta	2.521.005	694 (13,0)	12,2 (10,7–15,6)
Outras	315.288	103 (2,0)	1,6 (1,0–2,7)
Programa Bolsa Família^c^			
Não	14.560.610	3.627 (68,0)	74,6 (69,6–79,1)
Sim	3.920.342	1.428 (27,0)	20,1 (16,6–24,1)
Não sabe/não respondeu	1.035.610	283 (5,0)	5,3 (2,8–9,8)
Tipo de serviço procurado			
Serviço público	9.244.010	2.687 (50,0)	47,4 (41,9–52,9)
Serviços particulares	10.272.553	2.651 (40,0)	52,6 (47,1–58,1)
Escolaridade			
Ensino fundamental incompleto	2.644.193	734 (14,0)	13,5 (11,2–16,2)
Ensino fundamental completo e médio incompleto	3.841.540	891 (17,0)	19,7 (15,7–24,3)
Ensino médio completo e ensino superior incompleto	9.069.458	2.450 (46,0)	46,5 (40,7–52,4)
Ensino superior completo	3.961.372	1.263 (24,0)	20,3 (16,8–24,3)
Renda familiar em salários mínimos (SM)			
Sem informação	4.554.184	1.348 (25,0)	23,3 (18,4–29,1)
Até 1 SM	3.691.039	981 (18,0)	18,9 (14,8–23,9)
> 1 a 3 SM	6.827.223	1.829 (34,0)	35,0 (30,0–40,3)
> 3 SM	4.444.117	1.180 (22,0)	22,8 (19,0–27,0)
**Variáveis municipais**			
Porte demográfico			
Até 20 mil		69 (21,5)	12,8 (8,4–19,1)
> 20 a 50 mil		69 (21,5)	12,5 (8,5–18)
> 50 a 100 mil		56 (17,5)	10,6 (6,8–16,1)
> 100 mil		126 (39,5)	64,1 (56,0–71,4)
Nível de desenvolvimento socioeconômico			
Crítico (< 0,4)		13 (4,0)	1,2 (0,5–2,8)
Baixo (0,4 a < 0,6)		128 (40,0)	23,4 (18,2–29,6)
Médio (0,6 a < 0,8)		145 (45,4)	52,0 (42,6–61,2)
Alto (≥ 0,8)		34 (10,6)	23,4 (16,7–31,8)
Cobertura potencial de saúde bucal na AB			
< 40%		53 (16,6)	30,9 (21,9–41,8)
40% a < 60%		64 (20,0)	29,8 (22,9–37,9)
≥ 60%		203 (63,4)	39,2 (31,6–47,4)

IC95%: intervalo de confiança de 95%; AB: atenção básica.

Nota: variável Programa Bolsa Família referente à adesão no programa de transferência de renda governamental.

^a^ Equivalente à estimativa populacional.

^b^ Amostra do estudo SB Brasil 2023 (%).

^c^ Programa governamental de transferência condicionada de renda.

Na população do estudo, houve equilíbrio entre as faixas etárias de 35 a 39 (51,9%; IC95% 47,8–56,0) e 40 a 44 (48,1%; IC95% 44,0–52,1). Predominaram mulheres (70,1%; IC95% 66,5–73,4), adultos autodeclarados brancos (43,1%; IC95% 39,0–47,3) e pardos (42,3%; IC95% 38,2–46,6) e que não recebiam auxílio governamental (74,6%; IC95% 69,6–79,1). Quase metade da amostra (46,5%; IC95% 40,7–52,4) possuía ensino médio completo ou superior incompleto, e em relação à renda familiar, 35,0% (IC95% 30,0–40,3) recebiam de um a três salários mínimos. Quanto ao tipo de serviço procurado, 47,4% (IC95% 41,9–52,9) demandaram um estabelecimento público e 52,6% (IC95% 47,1–58,1) um serviço privado.

Na Tabela 2, é mostrada a análise não ajustada. Níveis mais baixos de escolaridade e renda, participação do PBF e procura por serviço odontológico público mostraram-se significativamente associados a maior chance de falta de acesso aos serviços odontológicos. Adultos com ensino fundamental completo ou médio incompleto apresentaram quase três vezes mais chances de falta de acesso (OR = 2,87; IC95% 2,21–3,72; p < 0,000), em comparação àqueles com ensino superior completo. Valor próximo foi observado na comparação dos adultos com renda familiar de até um salário mínimo e aqueles com renda superior a três salários mínimos (OR = 2,36; IC95% 1,80–3,08; p = 0,000). Os indivíduos ligados ao PBF tiveram 26% mais chance de falta de acesso (OR = 1,26; IC95% 1,05–1,50; p = 0,009) em comparação aos não beneficiários. Os que procuraram por serviço odontológico público apresentaram mais que o dobro de chances de não obterem acesso (OR = 2,25; IC95% 1,91–2,66; p < 0,000), em comparação aos que buscaram serviços particulares. Não houve associação significativa entre o desfecho e as variáveis idade (p = 0,474), sexo (p = 0,816) e as categorias de cor/raça.

**Tabela 2:** Regressão logística multinível não ajustada para falta de acesso aos serviços odontológicos segundo as variáveis individuais e contextuais da população de adultos brasileiros de 35 a 44 anos de idade. Brasil, 2023 (5.338 adultos em 320 cidades).

Variáveis individuais	Procurou e não foi atendido/agendado				
	n	%	OR	IC95%	p
Sexo					
Masculino	237	16,8	Ref.		
Feminino	592	17,8	1,02	0,86–1,20	0,816
Idade					
35 a 39 anos	404	19,2	Ref.		
40 a 44 anos	425	15,7	1,05	0,90–1,23	0,474
Cor/raça					
Branca	251	18,3	Ref.		
Parda	446	17,4	1,07	0,89–1,29	0,495
Preta	112	14,7	1,16	0,90–1,50	0,261
Outras	20	20,6	1,41	0,83–2,39	0,204
Programa Bolsa Família					
Não	514	16,2	Ref.		
Sim	246	20,1	**1,26**	**1,05–1,50**	**0,009**
Não sabe/não respondeu	69	25,3	**1,70**	**1,23–2,34**	**0,001**
Tipo de serviço procurado					
Privado	274	16,2	Ref.		
Público	555	18,9	**2,25**	**1,91–2,66**	**< 0,000**
Escolaridade					
Ensino superior completo	115	8,8	Ref.		
Ensino médio completo e ensino superior incompleto	380	18,6	**1,85**	**1,47–2,32**	**< 0,000**
Ensino fundamental completo e médio incompleto	197	23,5	**2,87**	**2,21–3,72**	**< 0,000**
Ensino fundamental incompleto	137	18	**2,55**	**1,92–3,37**	**< 0,000**
Renda familiar em salários mínimos (SM)					
> 3 SM	117	10,4	Ref.		
> 1 a 3 SM	285	13,5	**1,65**	**1,30–2,10**	**< 0,000**
Até 1 SM	188	26,7	**2,36**	**1,80–3,08**	**< 0,000**
Sem informação	239	22,9	**1,88**	**1,46–2,42**	**< 0,000**
**Variáveis municipais**					
Porte demográfico					
> 100 mil	126	16,1	Ref.		
> 50 a 100 mil	56	17,6	1,13	0,70–1,82	0,604
> 20 a 50 mil	69	17,5	1,24	0,82–1,88	0,290
Até 20 mil	69	24,2	1,34	0,87–2,06	0,175
Nível de desenvolvimento socioeconômico					
Alto (≥ 0,8)	34	12,2	Ref.		
Médio (0,6 a < 0,8)	145	19,5	1,47	0,89–2,43	0,126
Baixo (0,4 a < 0,6)	128	17,4	1,49	0,88–2,54	0,135
Critico (< 0,4)	13	33,6	1,91	0,77–4,75	0,158
Cobertura potencial de saúde bucal na AB					
≥ 60%	203	19,5	Ref.		
40% a < 60%	64	9,7	**0,68**	**0,47–0,98**	**0,040**
< 40%	53	22,4	1,05	0,70–1,58	0,779

OR: *odds ratio*; IC95%: intervalo de confiança de 95%; AB: atenção básica.

Nota: variável Programa Bolsa Família refere-se ao programa governamental de transferência condicionada de renda. Valores em negrito representam associações significativas.

Em relação às variáveis contextuais, apenas a cobertura de saúde bucal na atenção básica entre 40,0% e 60,0% mostrou-se associada a uma menor chance de falta de acesso (OR = 0,68; IC95% 0,47–0,98; p = 0,040) em comparação à cobertura igual ou superior a 60,0%. As demais variáveis contextuais não apresentaram associações estatisticamente significativas (Tabela 2). Investigando as características dos municípios de residência da população do estudo, observou-se significativa associação entre municípios com mais de 100 mil habitantes e coberturas da saúde bucal na atenção básica inferiores a 60,0% (p < 0,000), e municípios com baixo e crítico desenvolvimento e coberturas de 60,0% ou mais da saúde bucal na atenção básica (p < 0,000) (dados não mostrados na Tabela 2).

No modelo multinível ajustado (Tabela 3), permaneceram significativas as associações entre a falta de acesso, escolaridade, renda familiar e tipo de serviço procurado, mesmo após o ajuste por sexo e idade (modelo 1). Adultos com ensino fundamental incompleto apresentaram 1,75 (IC95% 1,28–2,38) vez mais chances de não obter acesso aos serviços odontológicos, em comparação aos indivíduos com ensino superior completo. Essa chance foi 2,10 (IC95% 1,59–2,79) vezes maior para os adultos com ensino fundamental completo e médio incompleto. Quanto à renda, indivíduos que possuíam renda familiar menor que um salário mínimo apresentaram 1,51 (IC95% 1,12–2,04) vez mais chances de experimentar a falta de acesso. Aqueles adultos que procuraram por serviços odontológicos públicos tiveram quase duas vezes mais chances de não obter acesso (OR = 1,90; IC95% 1,59–2,26).

**Tabela 3:** Regressão logística multinível ajustada para falta de acesso aos serviços odontológicos segundo as variáveis individuais e contextuais da população de adultos brasileiros de 35 a 44 anos de idade. Brasil, 2023 (5.338 adultos em 320 cidades).

Efeitos fixos	Modelo 1^a^	Modelo 2^a^	Modelo 3^a^	Modelo 4^a^
Variáveis individuais	OR (IC95%)	OR (IC95%)	OR (IC95%)	OR (IC95%)
Programa Bolsa Família				
Não	Ref.	Ref.	Ref.	Ref.
Sim	0,88 (0,73–1,07)	0,88 (0,73–1,07)	0,90 (0,73–1,08)	0,89 (0,73–1,08)
Não sabe/não respondeu	1,26 (0,90–1,77)	1,26 (0,90–1,78)	1,26 (0,89–1,77)	1,26 (0,89–1,77)
Tipo de serviço procurado				
Privado	Ref.	Ref.	Ref.	Ref.
Público	**1,90 (1,60–2,26)**	**1,90 (1,59–2,26)**	**1,90 (1,58–2,27)**	**1,92 (1,61–2,28)**
Escolaridade				
Superior completo	Ref.	Ref.	Ref.	Ref.
Médio completo e ensino superior incompleto	**1,46 (1,15–1.87)**	**1,47 (1,16–1,88)**	**1,47 (1,15–1,87)**	**1,46 (1,15–1,87)**
Fundamental completo e médio incompleto	**2,12 (1,60–2,82)**	**2,14 (1,61–2,83)**	**2,13 (1,61–2,83)**	**2,13 (1,61–2,82)**
Fundamental incompleto	**1,75 (1,28–2,39)**	**1,75 (1,28–2,38)**	**1,76 (1,29–2,40)**	**1,76 (1,29–2,40)**
Renda familiar em salários mínimos				
> 3 SM	Ref.	Ref.	Ref.	Ref.
> 1 a 3 SM	1.22 (0,94–1,58)	1,22 (0,94–1,57)	1,22 (0,94–1,57)	1,23 (0,95–1,58)
Até 1 SM	**1,52 (1,13–2.05)**	**1,51 (1,12–2,04)**	**1,51 (1,12–2,03)**	**1,52 (1,13–2,05)**
Sem informação	**1,35 (1,02–1,77)**	**1,35 (1,03–1,77)**	**1,35 (1,02–1,77)**	**1,33 (1,01–1,75)**
**Variáveis municipais**				
Porte demográfico				
>100 mil		Ref.		
> 50 a 100 mil		1,11 (0,69–1,78)		
> 20 a 50 mil		1,21 (0,80–1,83)		
Até 20 mil		1,14 (0,74–1,74)		
Nível de desenvolvimento socioeconômico				
Alto (≥ 0,8)			Ref.	
Médio (0,6 a < 0,8)			1,34 (0,82–2,18)	
Baixo (0,4 a < 0,6)			1,21 (0,71–2,05)	
Crítico (< 0,4)			1,52 (0,61–3,75)	
Cobertura potencial de saúde bucal na AB				
≥ 60%				Ref.
40% a < 60%				0,73 (0,51–1,04)
< 40%				1,22 (0,81–1,83)
Efeitos aleatórios	Estimativas			
Variância (erro padrão)	0,405 (0,150)	0,390 (0,146)	0,409 (0,150)	0,420 (0,148)
Modelo vazio 0,483 (0,163)				
Porcentagem da variância explicada	16,1%	3,2%	-0,8%	-3,1%
Teste da razão de verossimilhança	< 0,000	< 0,000	< 0,000	< 0,000
CCI	16,1	19,3	15,3	13,0

OR: *odds ratio*; IC95%: intervalo de confiança de 95%; AB: atenção básica; SM: salário mínimo; CCI: coeficiente de correlação intraclasse.

Nota: CCI que corresponde à porcentagem da variância total do nível 2 explicada pelo modelo; variável Programa Bolsa Família referente à adesão no programa de transferência de renda governamental. Valores em negrito representam associações significativas

^a^ Ajustadas pelas variáveis demográficas (sexo, idade e cor/raça).

Entre as variáveis municipais, não se observaram associações significativas no modelo final. Os valores de OR para os municípios com cobertura intermediária de saúde bucal na atenção básica (de 40,0% a 60,0%) perderam significância estatística após o ajuste pelas variáveis individuais (OR = 0,73; IC95% 0,51–1,04; p = 0,079) em comparação aos municípios com cobertura mais elevada (maior ou igual a 60,0%). A variância entre os municípios estudados reduziu de 0,48 no modelo nulo para 0,41 no Modelo 1 e 0,39 no Modelo 2. A inclusão do porte demográfico explicou 19,3% da variância total.

## DISCUSSÃO

Os resultados mostraram que a condição socioeconômica, expressa por níveis baixos de escolaridade e de renda familiar, e a busca por serviço público foram significativamente associadas à falta de acesso aos serviços odontológicos entre os adultos brasileiros, considerando sua distribuição entre as cidades, após o ajuste por fatores sociodemográficos e as características dos municípios de residência relacionadas ao porte populacional, ao desenvolvimento socioeconômico e à cobertura potencial de serviços odontológicos na rede de atenção básica à saúde. Os fatores contextuais não explicaram o desfecho, adicionalmente aos fatores de ordem individual. Este resultado também é relevante porque é compatível com a hipótese de capilaridade dos serviços odontológicos nos diferentes municípios do país.

Diferentemente da literatura acerca das características ligadas à utilização dos serviços de saúde, há uma carência de estudos que investiguem os fatores associados ao acesso a esses serviços^6^. A multiplicidade de definições e interpretações do termo e a frequente aplicação do conceito de utilização como *proxy* de acesso podem estar conectadas a essa lacuna. Com enfoque maior nas características individuais, Nunes et al.^18^ observaram que a falta de acesso foi mais frequente em indivíduos com menor renda, mas o mesmo padrão não foi observado quando avaliada a utilização dos serviços. Este achado serve de alerta para as diferenças conceituais entre os termos e reforça a importância de estudos que tratem o acesso como um fenômeno distinto e multifatorial, como propõe este estudo.

Os resultados desfavoráveis aos adultos com menor renda e escolaridade foram compatíveis com dois estudos brasileiros de base populacional^11,19^. As discrepâncias observadas referem-se às estimativas de falta de acesso e podem ser atribuídas principalmente às diferenças de idade da população considerada e do indicador utilizado. Enquanto no presente estudo, o valor foi 17,5% entre os adultos de 35 a 44 anos de idade que procuraram e não foram atendidos/agendados nos últimos 12 meses, na pesquisa utilizando dados de um inquérito telefônico do ano de 2009, reportou-se o valor de 15,2% considerando o mesmo indicador, mas a população com mais de 18 anos de idade residente nas 26 capitais e o Distrito Federal^19^, e no estudo empregando dados da Pesquisa Nacional por Amostra de Domicílios para os anos 2003 e 2008, relataram-se, respectivamente, valores de 4,5% e 3,3%, considerando somente as duas últimas semanas e todas as idades^11^.

Chaves et al.^20^ e Carreiro et al.^21^ investigaram a falta de acesso aos serviços odontológicos em municípios dos estados da Bahia e Minas Gerais, respectivamente. A falta de acesso foi relatada por 15,2% dos indivíduos nos municípios baianos e por 10,3% no município mineiro^21^. Enquanto para Chaves et al.^20^, o acesso foi investigado como sinônimo de acessibilidade, ou seja, características ligadas aos serviços que facilitam sua utilização pelos usuários, Carreiro et al.^21^ investigaram o acesso como a obtenção de atendimento em resposta à procura de assistência pelo indivíduo.

De acordo com Almeida et al.^6^, sistemas de saúde universais, assim como o SUS, encerram grande potencialidade para a redução das iniquidades no acesso aos serviços de saúde. Contudo, no presente estudo, observou-se maior possibilidade de falta de acesso entre os adultos que procuraram o serviço público. Pode-se levantar a hipótese de que, quando os adultos buscam atendimento odontológico em serviços públicos, ficam expostos a serviços que apresentam características organizacionais incompatíveis com suas necessidades (turnos e/ou horários de funcionamento incompatíveis, filas extensas, baixa resolutividade, localização geográfica de difícil acesso)^22^. Nesse sentido, Chaves et al.^20^ recomendaram algumas estratégias de marcação de consulta, que poderiam contribuir para a substituição dos indivíduos faltosos e a diminuição de filas para atendimento. Além disso, estudo realizado em mais de 18 mil unidades públicas no ano de 2014 mostrou que somente 14,8% delas tinham todo o material necessário para realizar os procedimentos odontológicos da atenção básica^23^. Para esse mesmo ano, havia um dentista vinculado ao SUS para cerca de quatro mil pessoas, enquanto no setor privado, a relação era de 1 para 649 habitantes^24^.

Para além destes fatores relacionados ao sistema público de saúde, as condições socioeconômicas e estruturais dos territórios onde se localizam tais serviços também poderiam estar associadas ao acesso da população adulta^17,25^. Fatores ligados aos meios de transporte e à baixa disponibilidade dos serviços odontológicos em determinadas localidades poderiam atuar como barreiras limitantes no acesso a esses serviços^26^. Entretanto, os resultados observados foram compatíveis com a hipótese de capilaridade dos serviços odontológicos nos diferentes municípios do país, ou seja, a heterogeneidade que caracteriza os municípios segundo o porte demográfico, o desenvolvimento socioeconômico e a provisão de serviços públicos odontológicos de atenção básica não foram elevados o suficiente para refletir diferenças relevantes no acesso dos adultos brasileiros.

Entre as limitações do estudo, poderia ter sido arguida a presença na análise das variáveis PBF e renda familiar com dados faltantes. Como são escassas as investigações sobre fatores contextuais ligados ao acesso, e a remoção dessas observações poderia reduzir o número de cidades incluídas, a opção foi por manter essas observações como uma categoria isolada a fim de preservar a variedade de observações de nível 2 e a interpretabilidade dos achados. O tipo de medida de acesso representada pelo autorrelato poderia ser visto como uma limitação se o foco do estudo fosse dirigido para as características da oferta de serviços odontológicos. Entretanto, como o foco foi investigar o acesso do ponto de vista da experiência da população adulta urbana, a entrevista domiciliar pode ser considerada mais apropriada como técnica de coleta de dados. Acresce-se o rigor metodológico e a validação do instrumento que caracterizaram a pesquisa^13^. O desenho do estudo não permite interpretar as associações observadas como relações de causa-efeito nem extrapolar as observações para a população adulta residente em área rural. A determinação de tais relações requer outros tipos de estudos e de variáveis. Por exemplo, estudos que incluam variáveis organizacionais ligadas às características de oferta dos serviços podem contribuir para elucidar o papel dessas características para facilitar ou dificultar o acesso da população adulta. Em que pesem esses aspectos, cabe destacar a relevância deste estudo ao analisar a falta de acesso aos serviços odontológicos, definida como a não obtenção de atendimento após a identificação da necessidade e a busca por serviço, com base em uma amostra complexa de base populacional proveniente da Pesquisa Nacional SB Brasil 2023. Ainda que o conceito possa assumir diferentes acepções na literatura científica, um aspecto comum é que o acesso, seja a um serviço, a um provedor de serviços ou a uma instituição, é definido em referência às necessidades do indivíduo^1^. Outro ponto forte a ser mencionado foi a adoção da análise multinível, que possibilitou compreender o acesso aos serviços odontológicos a partir da interação entre determinantes individuais e contextuais, reconhecendo características socioeconômicas e territoriais dos municípios, uma perspectiva pouco investigada.

Em conclusão, a falta de acesso aos serviços odontológicos em adultos mostrou associação com a baixa condição socioeconômica e com a procura pelo serviço público, considerando as diferenças entre os municípios relacionadas ao porte populacional, ao desenvolvimento e à cobertura potencial de serviços odontológicos na rede de atenção básica à saúde. A ausência de associação entre as variáveis contextuais analisadas e a falta de acesso pode refletir a capilaridade dos serviços odontológicos, um aspecto extremamente positivo do sistema de saúde brasileiro. Ainda assim, a despeito dos avanços na ampliação da cobertura e da instituição da Política Nacional de Saúde Bucal como política de Estado, persistem desigualdades socioeconômicas que limitam o princípio da equidade de acesso.
